# Genome-wide identification and expression analysis of the ADH gene family under diverse stresses in tobacco (*Nicotiana tabacum* L.)

**DOI:** 10.1186/s12864-023-09813-4

**Published:** 2024-01-02

**Authors:** Ruiqi Wang, Chaofan Du, Gang Gu, Binghui Zhang, Xiaolu Lin, Chengliang Chen, Tong Li, Rui Chen, Xiaofang Xie

**Affiliations:** 1 College of Life Sciences, Fujian Agriculture & Forestry University, Fuzhou, 350002 China; 2 Longyan Tobacco Company, Longyan, 364000 China; 3 Institute of Tobacco Science, Fujian Provincial Tobacco Company, Fuzhou, 350003 China; 4 Jianning Branch of Sanming Tobacco Company, Sanming, 354500 China; 5 Fujian Key Laboratory of Crop Breeding By Design, Fujian Agriculture & Forestry University, Fuzhou, 350002 China

**Keywords:** *Nicotiana tobacum* L., Alcohol dehydrogenases (ADH), Stress, Gene expression, Phylogenetic analysis

## Abstract

**Background:**

Alcohol dehydrogenases (ADHs) are the crucial enzymes that can convert ethanol into acetaldehyde. In tobacco, members of ADH gene family are involved in various stresses tolerance reactions, lipid metabolism and pathways related to plant development. It will be of great application significance to analyze the ADH gene family and expression profile under various stresses in tobacco.

**Results:**

A total of 53 ADH genes were identified in tobacco (*Nicotiana tabacum* L*.*) genome and were grouped into 6 subfamilies based on phylogenetic analysis. Gene structure (exon/intron) and protein motifs were highly conserved among the *NtADH* genes, especially the members within the same subfamily. A total of 5 gene pairs of tandem duplication, and 3 gene pairs of segmental duplication were identified based on the analysis of gene duplication events. *Cis*-regulatory elements of the *NtADH* promoters participated in cell development, plant hormones, environmental stress, and light responsiveness. The analysis of expression profile showed that *NtADH* genes were widely expressed in topping stress and leaf senescence. However, the expression patterns of different members appeared to be diverse. The qRT-PCR analysis of 13 *NtADH* genes displayed their differential expression pattern in response to the bacterial pathogen *Ralstonia solanacearum* L*.* infection. Metabolomics analysis revealed that *NtADH* genes were primarily associated with carbohydrate metabolism, and moreover, four *NtADH* genes (*NtADH20/24/48/51*) were notably involved in the pathway of alpha-linolenic acid metabolism which related to the up-regulation of 9-hydroxy-12-oxo-10(E), 15(Z)-octadecadienoic acid and 9-hydroxy-12-oxo-15(Z)-octadecenoic acid.

**Conclusion:**

The genome-wide identification, evolutionary analysis, expression profiling, and exploration of related metabolites and metabolic pathways associated with *NtADH* genes have yielded valuable insights into the roles of these genes in response to various stresses. Our results could provide a basis for functional analysis of *NtADH* gene family under stressful conditions.

**Supplementary Information:**

The online version contains supplementary material available at 10.1186/s12864-023-09813-4.

## Background

Alcohol dehydrogenase (ADH), also known as alcohol: NAD + oxidoreductase (EC 1.1.1.1), widely present in various organisms. ADH functions as a zinc-binding enzyme dimer that depends on NAD (P) co-factors to convert ethanol and acetaldehyde, and other pairs of short linear alcohols/aldehydes [1]. Each monomer comprises two primary structural domains: a substrate-binding or catalytic domain that includes an N-terminal region with irregular β-sheets and a short C-terminal region, and a coenzyme-binding domain, which has a double β-sheet segment called the Rossmann fold [2]. The ADH gene family is a vast family that includes three subfamilies: short-chain dehydrogenase/reductase (SDR)-ADH (∼250 amino acid residues), medium-chain dehydrogenase/reductase (MDR) (∼350 amino acid residues) and long-chain ADH or Iron-ADH gene superfamily (600∼750 amino acid residues or about 385∼900 amino acid residues) [3, 4]. Currently, the majority members of the ADH gene family identified in plants belong to the (MDR)-ADH subfamily, which usually have zinc ligands in their active site [5–7].

The ADH gene family plays vital roles in plant growth and development, as well as in responding to various stresses such as low temperature [8], drought [9], salt [10], mechanical damage [11], and the exogenous hormone abscisic acid [12]. In *Panax ginseng*, the positive response of *PgADHs* to abiotic stresses, including ABA, SA, and JA treatment, suggests that *PgADHs* are genuinely involved in these hormone-related stress responses [13]. In melon, *CmADHs* exhibit tissue-specific expression pattern, and play a role in response to various hormonal stresses [12]. Additionally, studies have shown that *ADH* genes play a key role in fruit ripening and aroma synthesis [7, 14, 15]. For example, the ADH enzyme activity of mango was enhanced with the increase of the *ADH* gene expression level at the initial stages of ripening [16]. A positive correlation has been identified between the expression level of *PbrADH*_*6*_ and both the total ADH activity and the production of volatile ester in pear [17]. The overexpression of *Le-ADH2* in mature tomato fruits alters the balance between certain alcohols and their corresponding aldehydes, which contribute to the formation of flavors, resulting in a stronger “ripe fruit” taste [18]. In addition, members of ADH gene family play a significant role in response to various pathogen infections. Studies have revealed that the silencing of *ADH* gene leads to a delayed hypersensitive response (HR) during non-host pathogen infections [19]. For instance, alcohol dehydrogenase 1 (*ADH1*) of barley acts as a susceptible gene (*S* genes) and it is involved in regulating the susceptibility to the fungus *Blumeria graminis f.sp.Hordei*. [20]. Typically, *S* genes have been considered as a source of broad-spectrum and durable resistance because the susceptibility factors encoded by *S* genes can be triggered during the infection process [21].

Tobacco is an important model plant. Several studies have demonstrated that the ADH family is widely involved in plant growth and development, fruit ripening, aroma volatilization and stress response [18, 22]. Currently, there is limited study on the members of ADH gene family in tobacco, and their exact functions remain unclear. Therefore, it is important to analyze the ADH gene family systematically in tobacco. The objectives of this study are to conduct a comprehensive investigation of ADH gene family, and investigate the expression patterns of ADH family members during different maturity stages and stress conditions by integrating tobacco transcriptome data. Additionally, our study aims to examine the changes of metabolites in the metabolic pathways regulated by the *ADH* genes under conditions of hypoxia stress and high-temperature curing. The information obtained from this study places an important foundation for further functional analysis on the ADH gene family and the trait improvement in tobacco.

## Results

### Characterization of *ADH* genes in tobacco genome

A total of 53 *NtADH* genes were identified in tobacco and were renamed from *NtADH1* to *NtADH53* (Table 1). Table 1 provides a detailed listing of information including gene ID, protein length (aa), molecular weight (MW), theoretical isoelectric point (pI), subcellular location and gene size. The results showed that the protein lengths varied greatly, with the longest tobacco ADH protein, NtADH1, comprising 1281 amino acid residues and the shortest, NtADH37, containing only 292 amino acid residues. The relative molecular weight ranged from 138.50 kDa (NtADH1) to 31.63 kDa (NtADH37). The theoretical isoelectric point (pI) varied from 9.36 (NtADH19) to 5.19 (NtADH38). Among them, a total of 24 NtADH proteins (45.3%) having a pI < 7 and 29 proteins (54.7%) exhibited a pI > 7. Analysis of the signal peptide revealed the absence of a conventional signal peptide at the N-terminal of all NtADHs. The subcellular localization prediction of NtADHs suggests that the majority of NtADHs may exist in the extracellular or cytoplasmic regions (Table 1), while a certain number of NtADHs may exist at nucleus, mitochondria or membrane bound chloroplast.

**Table1:** The information of *NtADH* genes in *Nicotiana tabacum*L

Gene Name	Gene identifier	Genomics position	Size (AA)	MW (Da)	PI	Subcellular location	Gene size
*NtADH1*	Nitab4.5_0000402g0150.1	Nt13	1281	138,504.24	6.39	Extracellular	30,680
*NtADH2*	Nitab4.5_0000496g0010.1	Nt13	413	44,261.68	8.16	Extracellular	7716
*NtADH3*	Nitab4.5_0002704g0020.1	Nitab4.5_0002704	414	44,489.05	8.6	Extracellular	7979
*NtADH4*	Nitab4.5_0001170g0200.1	Nt17	330	35,176.67	8.17	Cyto	3291
*NtADH5*	Nitab4.5_0003676g0090.1	Nitab4.5_0003676	330	35,133.68	7.71	Cyto	3308
*NtADH6*	Nitab4.5_0001054g0040.1	Nitab4.5_0001054	380	41,106.62	6.15	Cyto	2075
*NtADH7*	Nitab4.5_0001821g0060.1	Nitab4.5_0001821	380	41,176.66	6.2	Cyto	2083
*NtADH8*	Nitab4.5_0000385g0080.1	Nt22	398	43,109.97	8.12	Cyto	4650
*NtADH9*	Nitab4.5_0000262g0070.1	Nt13	387	41,305.64	8.9	Cyto	10,509
*NtADH10*	Nitab4.5_0000392g0070.1	Nt22	379	40,759.01	6.51	Cyto	4614
*NtADH11*	Nitab4.5_0007869g0030.1	Nitab4.5_0007869	388	42,023.52	6.13	Cyto	3321
*NtADH12*	Nitab4.5_0000445g0060.1	Nt12	381	41,420.46	6.03	Cyto	3629
*NtADH13*	Nitab4.5_0017191g0010.1	Nitab4.5_0017191	386	41,086.33	8.57	Cyto	5022
*NtADH14*	Nitab4.5_0000270g0270.1	Nt22	388	42,087.2	6.02	Cyto	3819
*NtADH15*	Nitab4.5_0001280g0030.1	Nitab4.5_0001280	367	40,092.67	6.16	Extracellular	1730
*NtADH16*	Nitab4.5_0005392g0080.1	Nitab4.5_0005392	357	38,795.64	5.75	Cyto	4757
*NtADH17*	Nitab4.5_0000103g0040.1	Nt04	349	37,307.48	9.35	Extracellular	4904
*NtADH18*	Nitab4.5_0002815g0020.1	Nt19	357	38,905.89	5.76	Cyto	4046
*NtADH19*	Nitab4.5_0001493g0030.1	Nitab4.5_0001493	351	37,475.64	9.36	Extracellular	7093
*NtADH20*	Nitab4.5_0000403g0140.1	Nt19	717	76,832.17	9.06	Extracellular	13,050
*NtADH21*	Nitab4.5_0000477g0170.1	Nt22	375	40,259.31	6.02	Cyto	4413
*NtADH22*	Nitab4.5_0010398g0010.1	Nitab4.5_0010398	385	41,432.82	6.17	Cyto	4343
*NtADH23*	Nitab4.5_0000402g0170.1	Nt13	360	39,178.27	6.21	Cyto	3290
*NtADH24*	Nitab4.5_0007704g0020.1	Nitab4.5_0007704	669	70,839.32	8.87	Extracellular	5179
*NtADH25*	Nitab4.5_0000402g0220.1	Nt13	348	37,739.56	6.82	Cyto	2611
*NtADH26*	Nitab4.5_0001539g0030.1	Nt06	383	42,029.64	5.65	Cyto	2971
*NtADH27*	Nitab4.5_0003503g0070.1	Nitab4.5_0003503	734	80,335.44	8.93	Extracellular	15,601
*NtADH28*	Nitab4.5_0001568g0140.1	Nitab4.5_0001568	327	35,210.96	7.02	Extracellular	3647
*NtADH29*	Nitab4.5_0001567g0060.1	Nitab4.5_0001567	311	34,436.22	7.02	Cyto	3785
*NtADH30*	Nitab4.5_0000402g0200.1	Nt13	359	38,908.95	6.59	Cyto	2460
*NtADH31*	Nitab4.5_0000964g0010.1	Nitab4.5_0000964	364	39,620.92	6.85	Cyto	4462
*NtADH32*	Nitab4.5_0001146g0200.1	Nt09	337	36,186.98	6.01	Extracellular	4140
*NtADH33*	Nitab4.5_0002520g0120.1	Nitab4.5_0002520	332	35,925.23	6.51	Cyto	3315
*NtADH34*	Nitab4.5_0000357g0320.1	Nt15	452	50,306.2	8.52	Cyto	7806
*NtADH35*	Nitab4.5_0005099g0030.1	Nitab4.5_0005099	376	40,633.8	6.59	Cyto	6494
*NtADH36*	Nitab4.5_0004465g0020.1	Nitab4.5_0004465	349	38,405.48	8.51	Mitochondrial/ Nucleus	11,103
*NtADH37*	Nitab4.5_0000487g0060.1	Nt13	292	31,627.21	7.57	Cyto	1601
*NtADH38*	Nitab4.5_0000588g0350.1	Nt12	301	32,788.7	5.19	Cyto	2282
*NtADH39*	Nitab4.5_0007051g0020.1	Nitab4.5_0007051	315	34,694.03	6.45	Cyto	2690
*NtADH40*	Nitab4.5_0000664g0350.1	Nt19	326	36,420.33	6.11	Cyto	5682
*NtADH41*	Nitab4.5_0004465g0010.1	Nitab4.5_0004465	354	38,836.82	7.07	Nucleus_and_Mitochondria	3874
*NtADH42*	Nitab4.5_0000402g0160.1	Nt13	347	38,011.76	5.96	Cyto	2109
*NtADH43*	Nitab4.5_0004465g0030.1	Nitab4.5_0004465	385	41,987.33	9.03	Nucleus_and_Mitochondria	6447
*NtADH44*	Nitab4.5_0003743g0020.1	Nitab4.5_0003743	385	42,036.49	9.14	Mitochondrial/Nucleus	4026
*NtADH45*	Nitab4.5_0003743g0010.1	Nitab4.5_0003743	342	37,652.48	8.42	Nucleus_and_Mitochondria	4433
*NtADH46*	Nitab4.5_0002473g0070.1	Nitab4.5_0003743	334	36,619.74	7.28	Cyto	5523
*NtADH47*	Nitab4.5_0007732g0050.1	Nitab4.5_0007732	989	108,658.15	7.96	Plasma membrane	17,666
*NtADH48*	Nitab4.5_0007704g0010.1	Nitab4.5_0002473	347	37,134.03	9.36	Extracellular	6559
*NtADH49*	Nitab4.5_0008803g0020.1	Nt20	395	42,419.83	9.21	Membrane bound Chloroplast	2443
*NtADH50*	Nitab4.5_0000203g0130.1	Nt24	379	40,351.32	7.03	Membrane bound Chloroplast	8143
*NtADH51*	Nitab4.5_0000403g0100.1	Nt19	347	37,024.89	9.05	Membrane bound Chloroplast	5385
*NtADH52*	Nitab4.5_0000332g0200.1	Nt21	377	40,725.99	9.04	Extracellular	7677
*NtADH53*	Nitab4.5_0003080g0140.1	Nitab4.5_0003080	535	58,283.92	9.32	Extracellular	9427

### Chromosome localization and collinearity analysis of *NtADH* genes

**Fig. 1:**
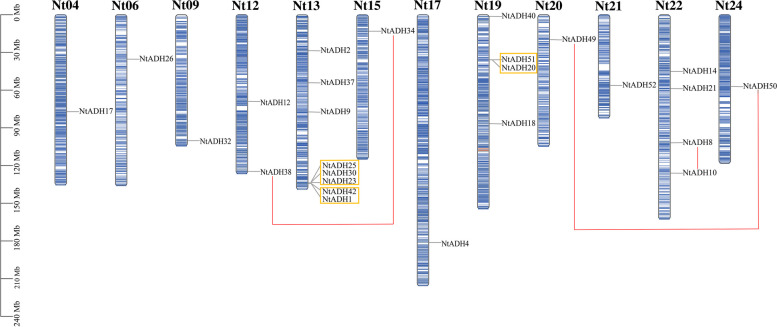
Chromosomal locations of *NtADH* genes. The scale on the left presents the length of chromosomes (Mb). Tandem duplicated gene pairs are displayed with boxes in yellow color, segmental duplicated gene pairs are connected by red lines

The analysis of chromosomal localization showed that some *NtADH* genes could not acquire the particular location due to the incomplete sequencing of the tobacco genome. Among the 53 *NtADH* genes, a total of 26 genes were unevenly distributed on 24 chromosomes of tobacco, while the remaining 27 *NtADH* genes were mapped to unassigned scaffolds (Fig. 1). The greatest number of *NtADH* genes were observed on chromosome 13, while chromosomes 19 and 22 had 4 *NtADHs* each. In contrast, chromosomes 4, 6, 9, 15, 17, 20, 21, and 24 had only one *NtADH* gene each. Moreover, it appears to be no relationship between chromosome size and quantity of gene that were identified. Five tandem duplication gene clusters were identified, including four on chromosome 13th (*NtADH23/NtADH25, NtADH23/NtADH30, NtADH25/NtADH30, NtADH1/NtADH42*), and one on chromosome 19th (*NtADH20/NtADH51*), while three segmental duplication gene pairs (*NtADH38/NtADH34*, *NtADH49/NtADH50*, *NtADH8/NtADH10*) were identified in this study (Fig. 1). The result implied that *NtADH* genes underwent gene duplication or loss during the evolution of tobacco genome.

**Fig. 2:**
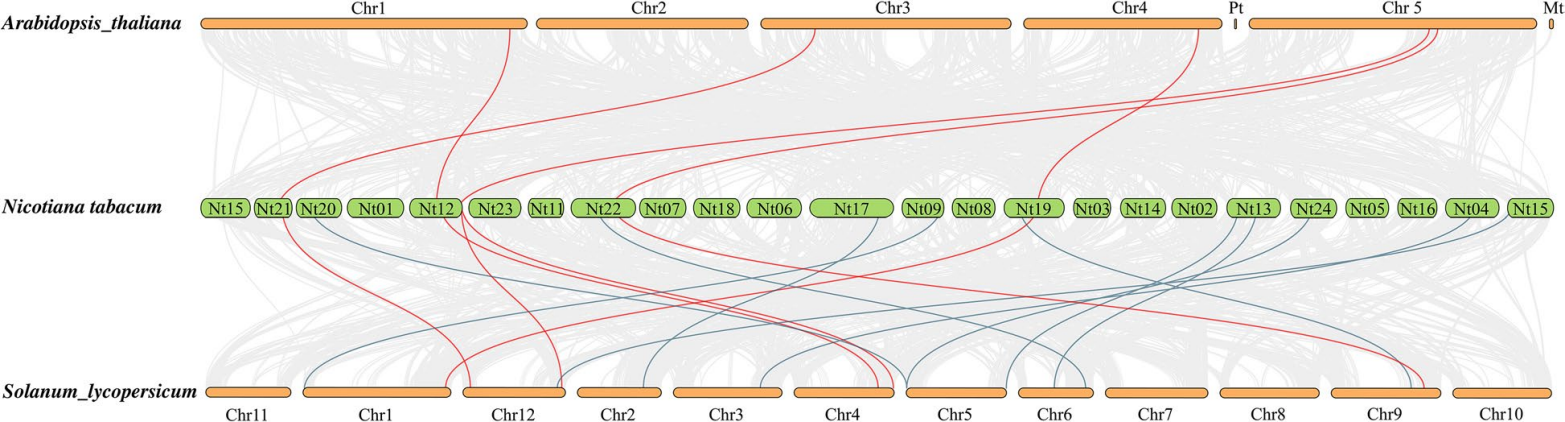
Syntenic analysis of ADH genes among tobacco, *Arabidopsis* and tomato. The *NtADH* gene (*NtADH8/12/18/38/52*) and its orthologous syntenic genes in *Arabidopsis* and tomato are linked by the red line. The syntenic *ADH* gene pairs located in the synteny blocks between tobacco and tomato are linked by blue lines

A total of 5 orthologous genes were identified between tobacco and *Arabidopsis* based on the interspecies syntenic analysis, while there are 16 syntenic counterparts between tobacco and tomato (Fig. 2). The genomic regions around *NtADH8*/*12/18/38/52* showed strong syntenic relationships with their counterparts in both *Arabidopsis* and tomato (Additional file 1: Table S1). Notably, good collinearity was detected among the *ADH* genes of three distinct species, even after undergoing speciation and long-term evolution, and the result suggested that these genes might have originated before solanaceae species diversification and retained conserved functional roles.

### Phylogenetics and gene structure analysis of *NtADHs*

**Fig. 3:**
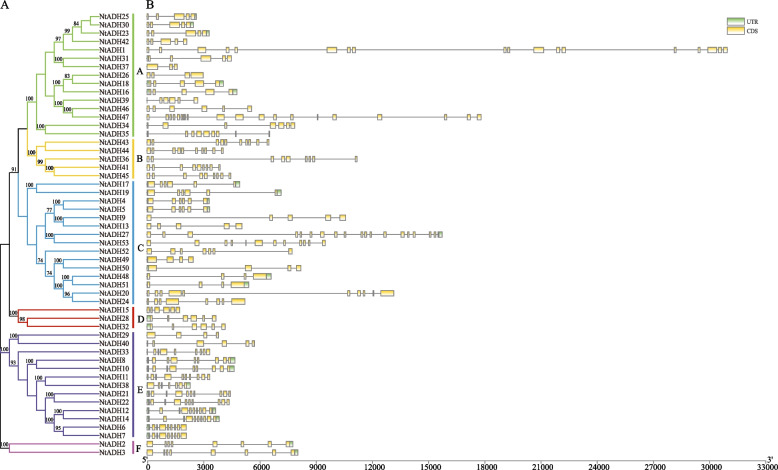
Gene structure and evolution of ADH family in *Nicotiana tabacum* L. **A** Phylogenetic relationships of *NtADHs*. Different subgroups were marked with different colors. **B** Intron–exon structure of *NtADHs*. Green boxes: UTR; Yellow boxes: CDS; spaces between the boxes: introns. The scale bar of bottom demonstrates the length of exons and introns

To investigate the evolutionary relationship between *NtADH* genes, a phylogenetic tree was constructed (Fig. 3A). The *NtADHs* were divided into 6 subgroups (A to F), with the largest members (15 members) found in subgroups A and C. These two subgroups represented more than 56.6% of the total *NtADH* members. In contrast, subgroups B, D and F had only 5, 3 and 2 members, respectively. Gene structure of *NtADHs* found that the number of exons was varied from 3 (*NtADH37*) to 20 (*NtADH47*) (Fig. 3B). Similar exon–intron structural patterns were observed among the *NtADH* members within the same subgroup, especially the number and length of exons. Within the 15 *NtADH* members clustered in subgroup A, 8 *NtADH* members were found to contain 5 exons and 4 introns. The members clustered in subgroup B had an average of 9.4 exons, with the highest average number of exons. Members clustered in group D contained an average of 6 exons, with the lowest number of exons. The number of introns in groups B, D and F is relatively conservative, while the number of introns in groups A, C and E is quite different. It can be deduced that the exon–intron structure of the family genes is intimately linked to evolution, and the expansion of family members is related to the insertion or deletion of introns.

### Domain and motif analysis of the NtADH proteins

**Fig. 4:**
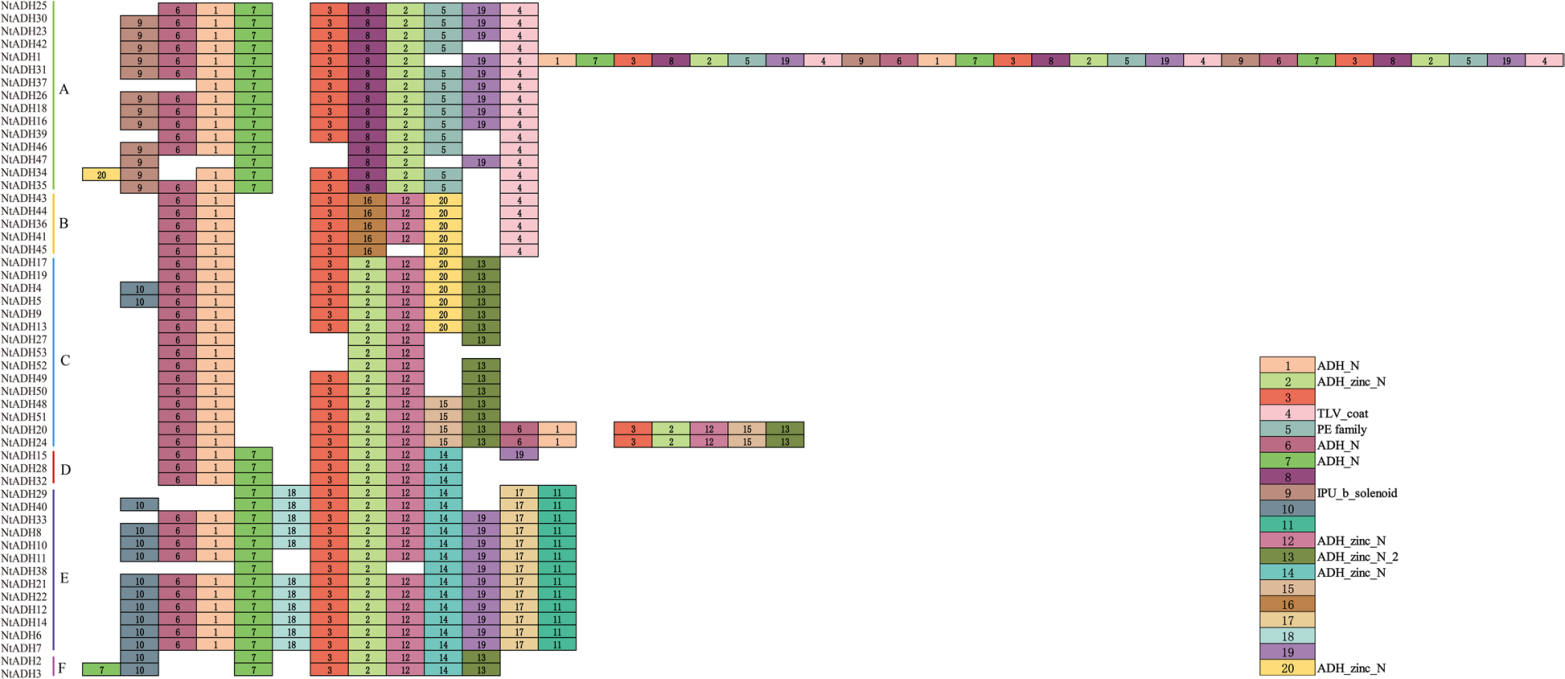
Conserved motifs for NtADH proteins in *Nicotiana tabacum* L. Different motifs are showed with different colored boxes and numbers (1–20)

A total of 20 conserved motifs have been identified and designated as motif1 to motif20 (Fig. 4). The conserved motifs presented within the same subgroup exhibited similar composition, indicating that the NtADH members clustered in the same subgroup may share similar biological functions. Most of the NtADH proteins were found to contain approximately 10 motifs, and there was no discernible correlation between the number of motifs and the length of the protein. For instance, despite having the shortest protein length, NtADH37 did not have the lowest number of motifs. In addition, different subgroups usually possessed specific motifs. For example, motif5 was exclusive to subgroup A while motif16 was exclusive to subgroup B. Likewise, motif11, motif17, and motif18 were solely presented in subgroup E, and motif17 and motif11 tended to appear in pairs.

**Fig. 5:**
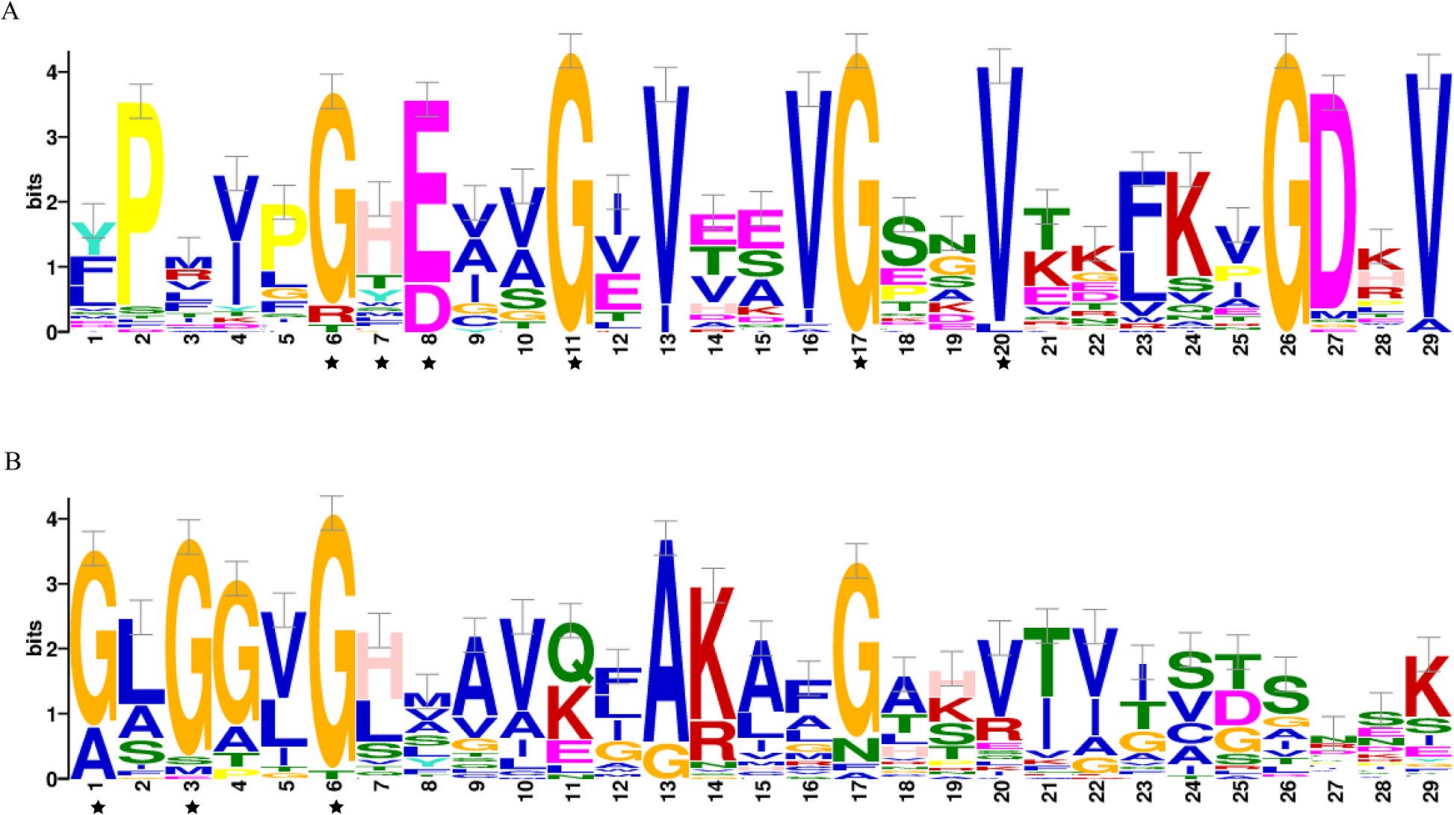
Sequence logos of the conserved ADH_N and ADH_zinc_N repeats of the NtADH domain. **A** Sequence logo of Zn1 in ADH_N. **B** Sequence logo of Rossman fold in ADH_zinc_N

The protein sequences of the 20 motifs were uploaded to CDD program for domain analysis (Fig. 4, Additional file 2: Table S2). Motif1, motif6, and motif7 were annotated as components of the GroES-like (ADH_N) domain, motif2, motif12, motif14, and motif20 were annotated as components of the zinc binding (ADH_zinc_N) domain. In addition, motif13, motif9, motif4 and motif5 were annotated as components of ADH_zinc_N_2 domain, IPU_b_solenoid, TLV_coat domain, and PE family, respectively. No annotation information was obtained for the remaining motifs. The result indicated that all members possessed the conserved regions of GroES-like (ADH_N) and zinc binding (ADH_zinc_N) domains. To further investigate the conservative domain of the NtADH proteins, the conserved domain of (ADH_N) and zinc binding (ADH_zinc_N) sequence logos of the 53 NtADH protein were generated by WebLogo (Fig. 5). The analysis revealed that NtADH members possess typical characteristics of ADH conserved domains and all the members had a Zn1 binding feature [GHE (X)2G (X)5G (X)2 V] (Fig. 5A) and a NADPH binding domain element [GXG (X)2G] (Fig. 5B). This result indicated that these proteins are likely to be zinc-dependent ADHs [23, 24].

### Phylogeny of plant ADH gene family

**Fig. 6:**
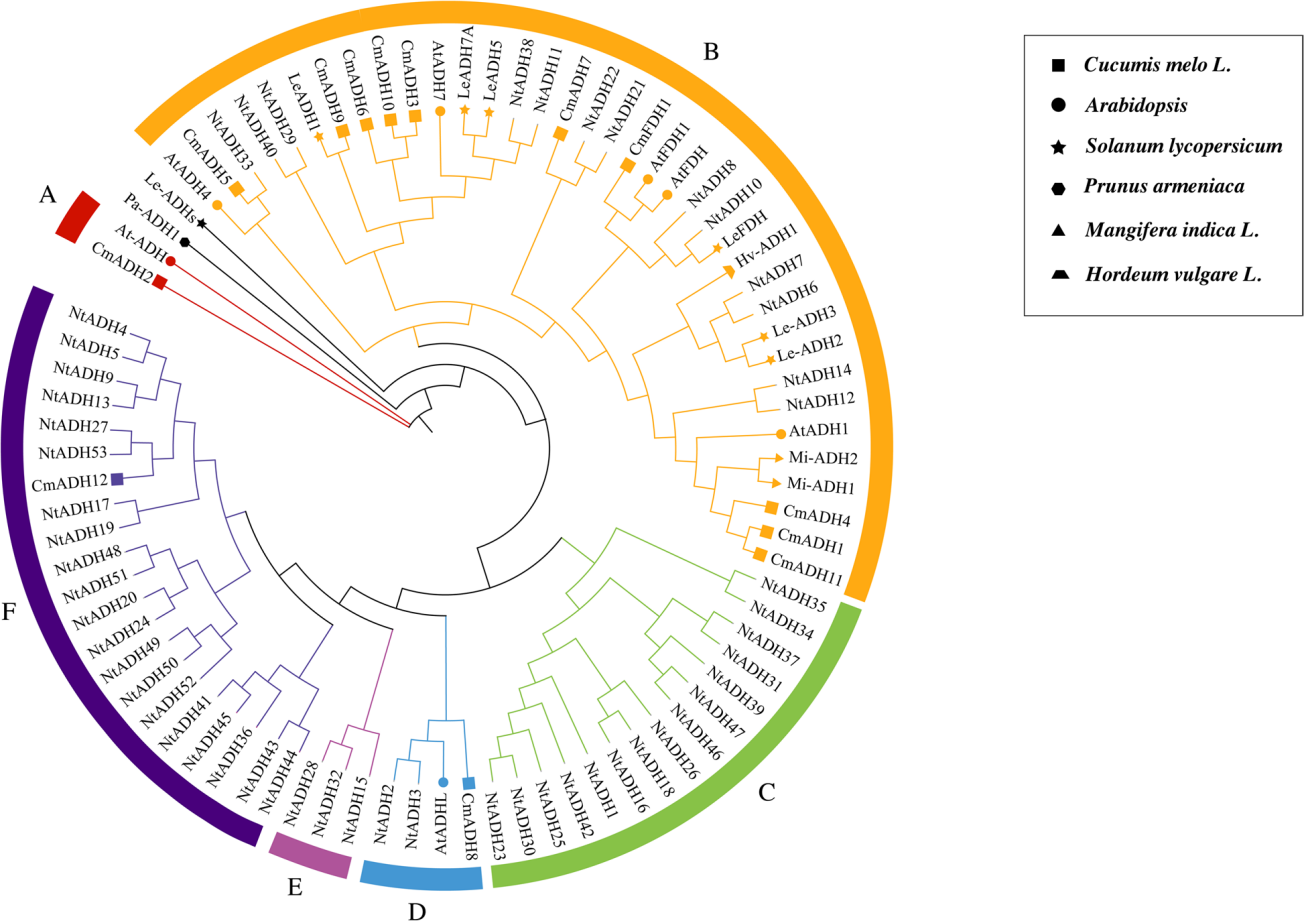
Phylogenetic tree of *Nicotiana tabacum*, melon, tomato, mango, apricot, *Arabidopsis* and barley ADH genes. The phylogenetic relationships were generated by using MEGA-11 using the Maximum Likelihood (ML) method (1000 bootstrap replicates). The squares, five-pointed star, triangle, hexagon, circles and trapezium represent melon, tomato, mango, apricot, *Arabidopsis* and barley ADH proteins, respectively

To explore the evolution of the ADH gene family, a total of 84 *ADH* gene members from 7 species were selected for the construction of phylogenetic tree (Fig. 6), including melons (13), *Arabidopsis thaliana* (7), apricot (1), mangos (2), tomato (7), barley (1) and tobacco (53) (Additional file 3: Table S3a). The ADH family members were clustered into 7 distinct subfamilies based on the phylogenetic tree, and the ADH members of tobacco were dispersed across 6 of these subfamilies, excluding subfamily A (short chain ADH protein). In addition, only *NtADH* members from tobacco were classified in subfamilies C, D and E. According to the phylogenetic tree, there were 31 sister pairs of homologous proteins, among which 4 pairs were orthologous genes and 27 pairs were paralogous (Additional file 3: Table S3b). Specifically, there were 21 paralogous pairs from tobacco, 2 pairs each from tomato and melon, and 1 pair each from mango and *Arabidopsis thaliana*. Previous studies have shown that CmADH1of melon [12], Mi-ADH of mango [16] and Le-ADH2 of tomato [18] are involved in the biosynthesis of fruit ripening and aroma volatiles, phylogenetic analysis showed that 13 *NtADH* genes (*NtADH33/40/29/38/11/22/21/8/10/7/6/14/12*) were grouped with these four genes (*CmADH1, Mi-ADH1/2, Le-ADH2*) suggesting that these *NtADH* genes may have a similar biological function and related to the biosynthesis of fruit ripening and aroma volatiles.

### *Cis*-acting regulatory elements analysis of *NtADH* genes

**Fig. 7:**
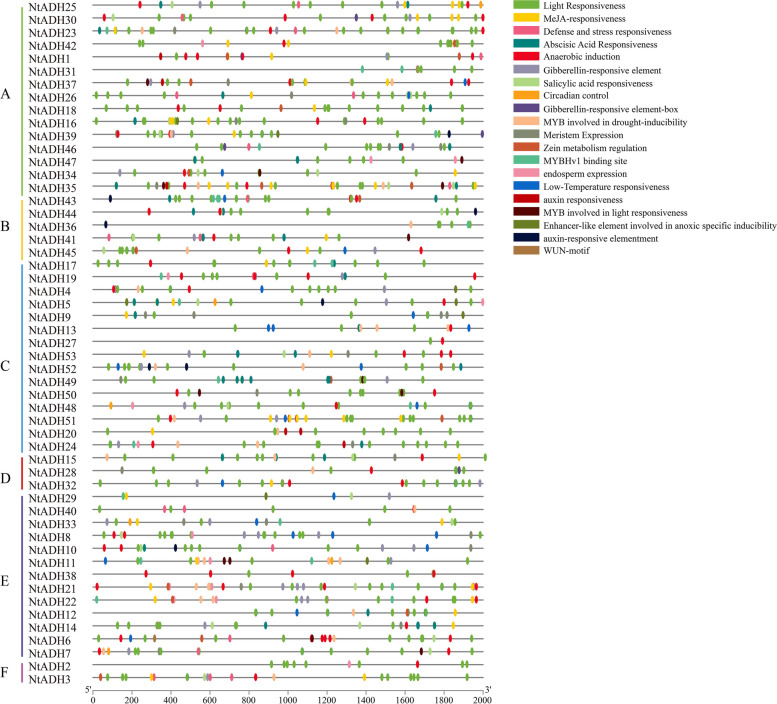
Predicted *cis*-elements in *NtADHs* promoters. Different shapes and colors represent the different types of *cis*-elements. Annotations of *cis*-elements were listed in Additional file 4: Table S4

The *cis*-elements in promoter regions play a critical role in the initiation of gene expression. A total of 58 *cis*- elements were selected in the *NtADH*s promoter region (Fig. 7). Among them, the light-responsive elements were the most common in the *NtADH* gene promoters, accounting for the largest proportion (42.28%), including G-box, Box 4, GT1 motif, and TCT motif. Meanwhile, there were many cis-regulatory elements that associated with phytohormone-responsive were also present, such as CGTCA-motifs, TGACG-motif, and ABRE. In addition, *cis-*regulatory elements that associated with the response to external or environmental stresses were also present, including stress response elements, ARE (cis-acting regulatory element essential for the anaerobic induction), MBS (MYB binding site involved in drought-inducibility), LTR (low-temperature response elements) and defense response elements TC-rich repeats (cis-acting regulatory element involved in defense and stress responsiveness). The result indicates that the expression of these *NtADH* genes is likely regulated by *cis*-elements associated with light-responsiveness, phytohormones, defense signaling transduction and various stresses during growth and development of tobacco.

### Expression analysis of *NtADH* genes under conditions of leaf senescence and topping stress

**Fig. 8:**
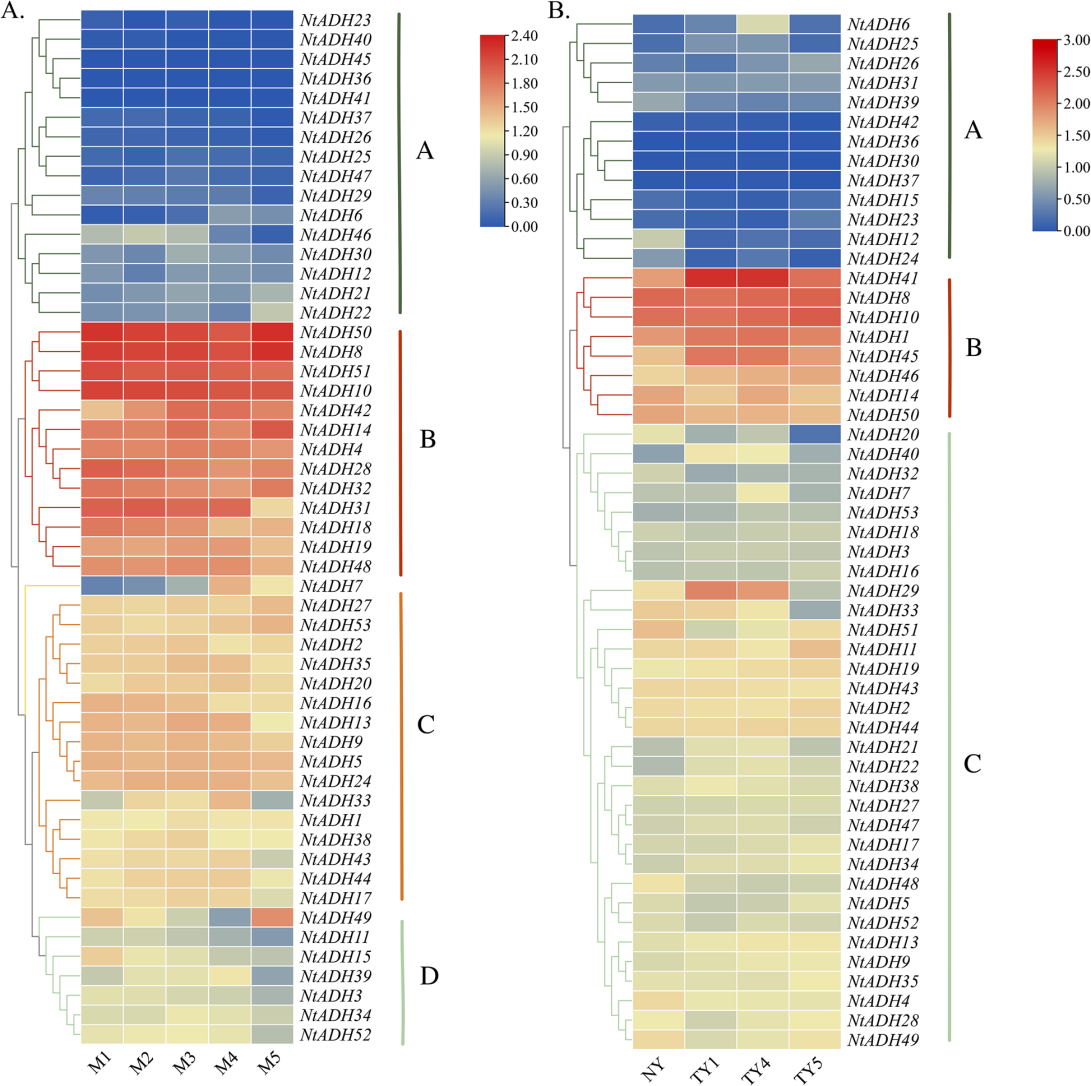
Gene expression profile under different condition **A** The expression of 53 *NtADHs* in tobacco leaves at five senescence stages. **B** The expression of 53 *NtADH* genes in response to topping. FPKM values for *NtADH* genes were transformed by log_10_(n + 1)

The FPKM values of *NtADH* genes at five senescence stages of tobacco leaves were obtained from our previous transcriptome data (Additional file 5: Table S5). Finally, the expression profiles of 53 *NtADH* genes were analyzed. The results showed that the members of *NtADH* genes had differential expression pattern in tobacco leaves at different senescence stages (Fig. 8A), and these 53 *NtADH* genes were clustered into four groups (A ~ D). A total of 13 *NtADH* genes were included in group B, and these genes had high expression level at the five senescence stages of leaves, implying that these genes could play important roles during leaves senescence process, while 16 *NtADH* genes clustered in group A showed a low or no expression during the whole senescence process. Notably, the expression levels of *NtADH7* genes increased gradually with the increasing of maturity, whereas those genes clustered in group D decreased in M5 stages except *NtADH49*. In terms of topping stress (Fig. 8B), the majority *NtADH* genes included in group B showed high expression levels at all stages, and some genes (*NtADH41/8/10/1/45/29*) had reached the peak expression level on the first and fourth days of topping, respectively. In contrast, the genes clustered in group A and C showed relative low expression level. These results indicated the functional diversity of tobacco *NtADH* members.

### Expression analysis of the *NtADHs* in response to *Ralstonia solanacearum*

**Fig. 9:**
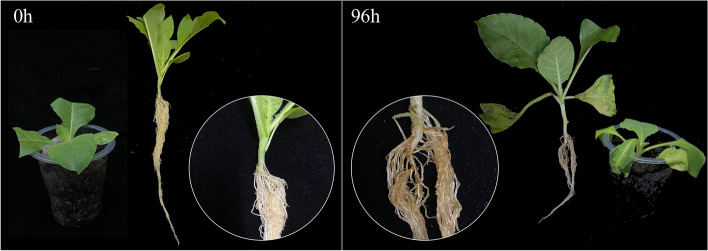
Disease symptoms in the cultivars Hongda at 0 h and 96 h by *Ras.* The basal parts of stems were magnified and shown in the circles. In 0 h (Left), and no symptoms were apparent. In 96 h (Right), leaves were withered and the basal part of stem was severely necrosis and turned to black

No obvious change was observed in the seedling at the initial stage after infected by *Ralstonia solanacearum* L. (*Ras*), however, the primary symptoms induced by *Ras* infection appeared in the seedling at 96 h (Fig. 9). At this stage, the seedling displayed leaf wilting and stem necrosis, while the roots turned yellowing and necrosis, whereas these symptoms were not apparent at 0 h (Fig. 9).

**Fig. 10:**
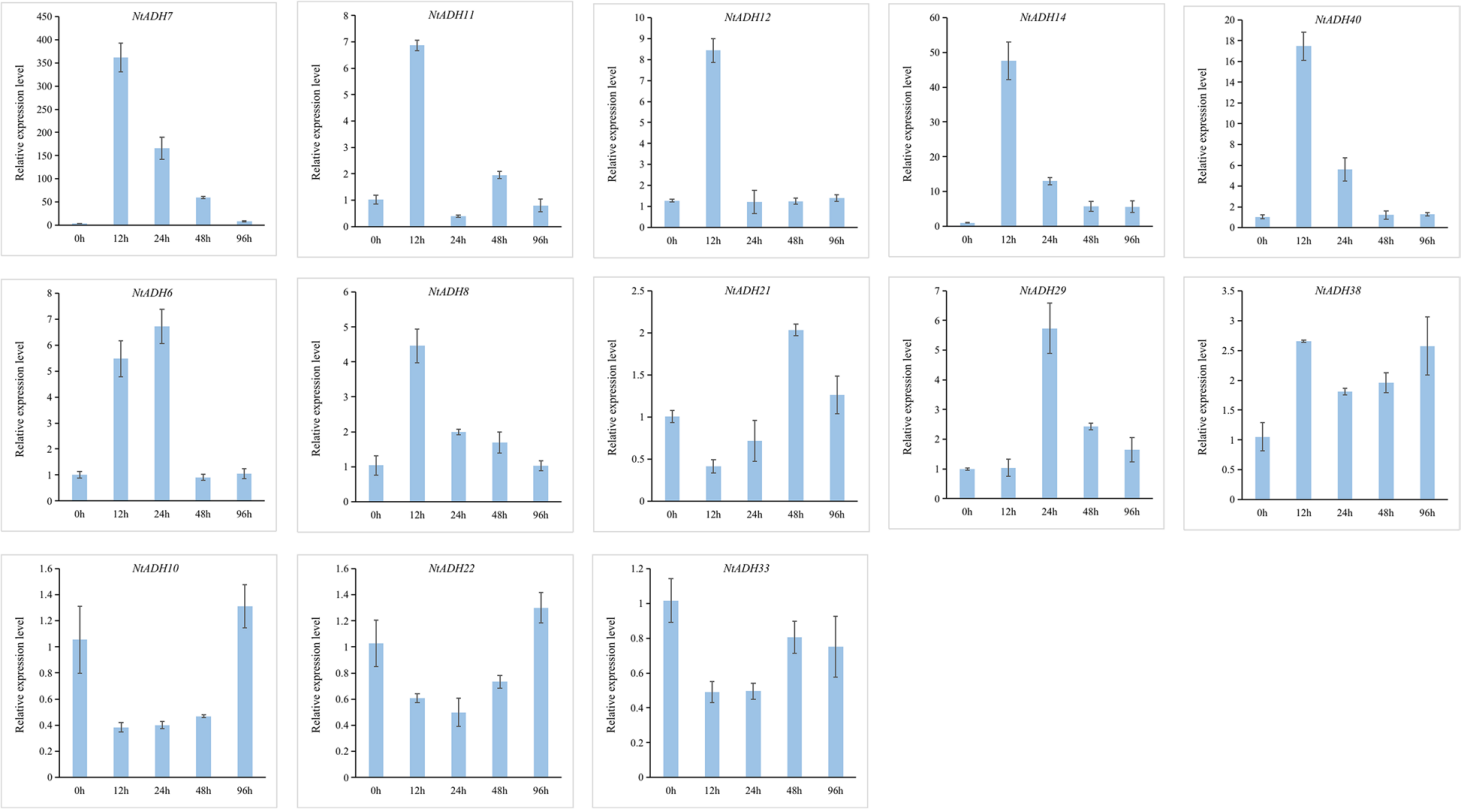
Relative expression level of 13 *NtADHs* in response to inoculation of *Ras*. Error bars are standard deviations of three biological replicates

To further explore the possible function of the *ADH* genes of tobacco, the expression patterns of *NtADHs* in response to pathogen infection were analyzed (Fig. 10). A total 13 tobacco *ADH* genes that clustered with *HvADH1* in B subgroups of the phylogenetic tree (Fig. 6) were selected for qRT-PCR analysis under *Ras* infection. Most of the selected genes displayed a notable up-regulated expression in response to the infection (Fig. 10). In comparison to the initial stage (0 h), a significant up-regulation was observed in 6 *NtADH* genes (*NtADH14*, *NtADH7*, *NtADH12*, *NtADH11*, *NtADH40*, *NtADH8*) at 12 h after inoculation. Specifically, *NtADH40* exhibited a remarkable up-regulation, surpassing a 15-fold increase, while *NtADH7* demonstrated an astonishing up-regulation of over 350-fold. While the expression of *NtADH6* and *NtADH29* increase significantly at 24 h after inoculation. In addition, the expression of 3 genes (*NtADH33*, *NtADH10* and *NtADH22*) displayed a gradual decrease in response to pathogen infection, followed by an increase. The expression patterns of the tobacco *ADH* genes in response to *Ras* infection revealed distinct variations in both response speed and intensity among the different genes.

### NtADH related-metabolomics analysis during hypoxia and high-temperature curing process in tobacco leaves

**Fig. 11:**
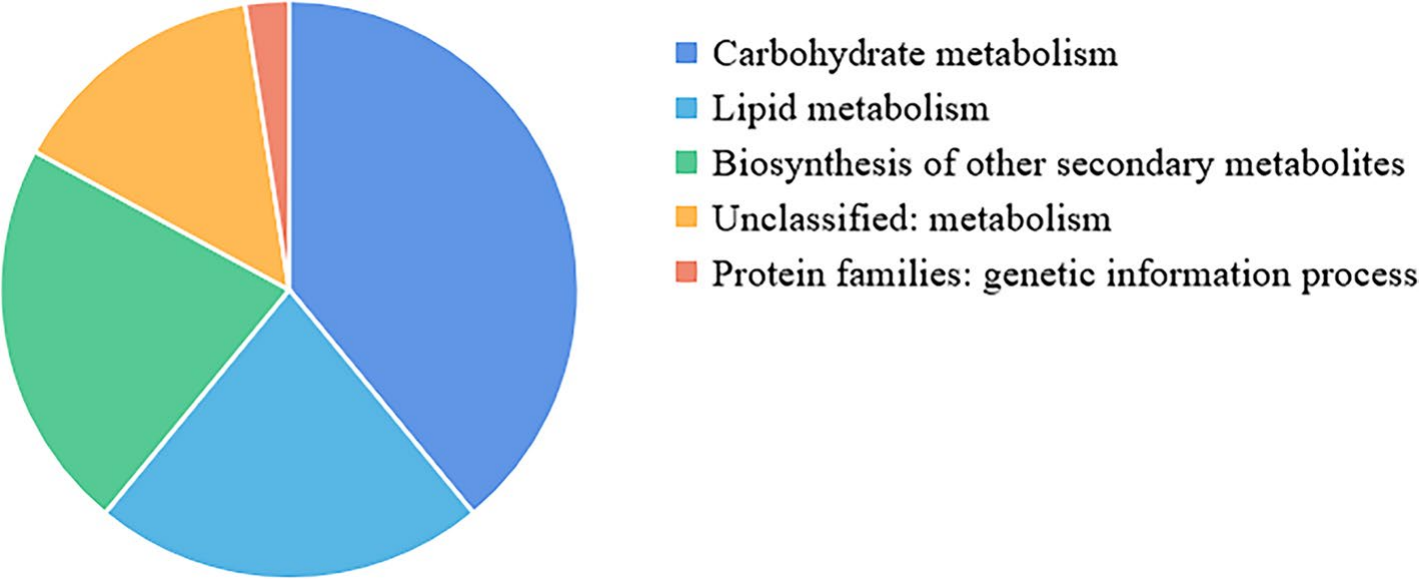
Metabolic pathways in which the ADH gene is involved

**Fig. 12:**
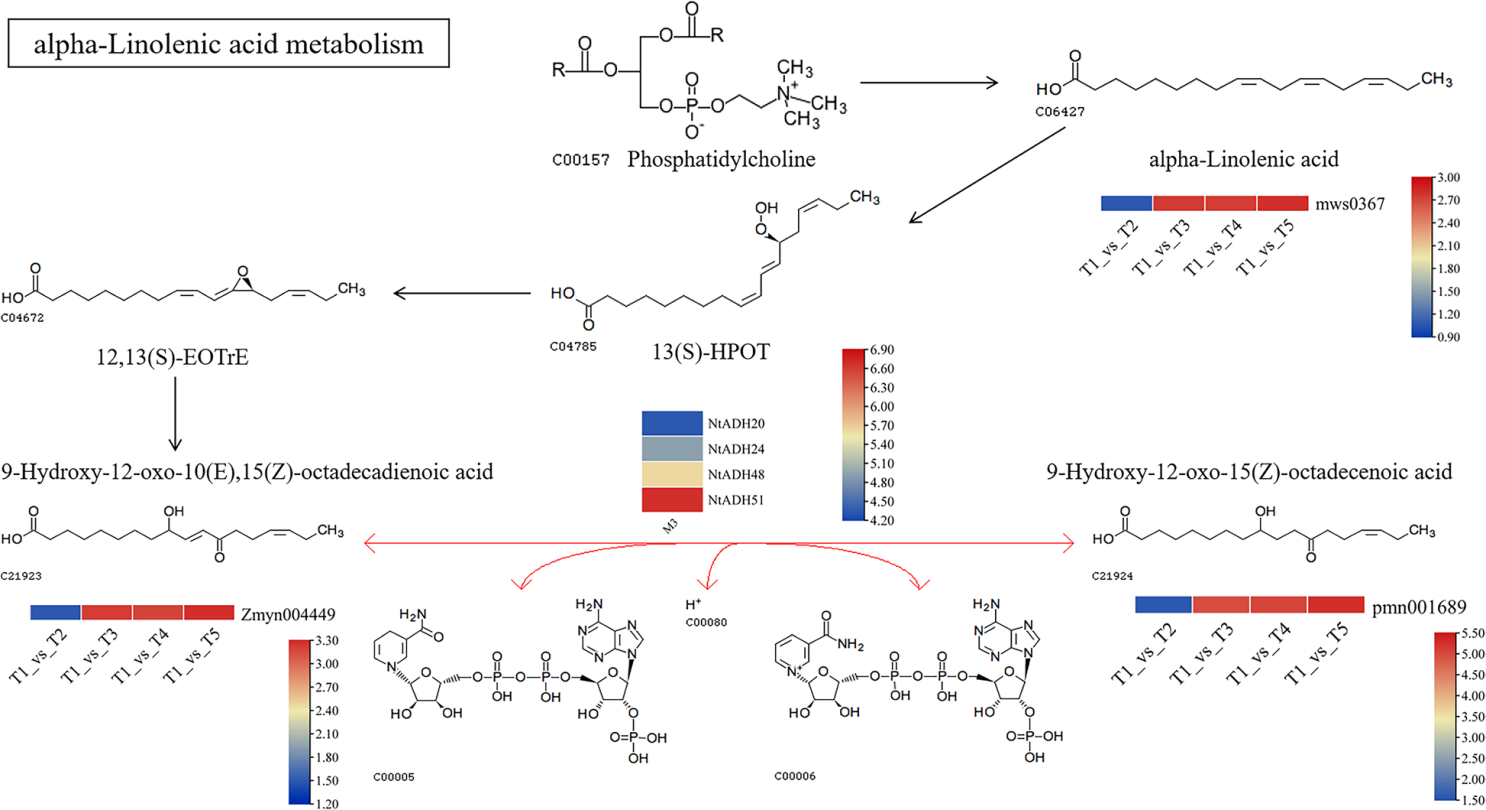
Overview of the alpha-linolenic acid pathways showing the changes of metabolite contents during curing process and the expression of *NtADHs* at the initial stage of curing

The *ADH* gene plays a crucial role in multiple metabolic processes. Among the 53 *NtADH* genes identified in tobacco, a total of 41 genes were annotated in the KEGG database, including carbohydrate metabolism, lipid metabolism, and the biosynthesis of other secondary metabolites (Fig. 11). During the hypoxia and high-temperature curing process, a total of 1129 metabolites were identified at four comparison pairs (Additional file 6: Table S6). Specifically, 137 differential metabolites were identified in the comparison of T1 VS T2, and 331 differential metabolites were identified in the comparison of T1 VS T3. In addition, 339 differential metabolites were identified in the comparison of T1 VS T4, and 322 differential metabolites were identified in the comparison of T1 VS T5. KEGG metabolite analysis indicated that these differential metabolites were enriched in the pathway of alpha-linolenic acid metabolism (ko00592), linoleic acid metabolism (ko00591), nucleotide metabolism (ko01232) and pyrimidine metabolism (ko00240), etc. (Additional file 7: Fig. 1 ~ 4). Among them, alpha-linolenic acid metabolism is the pathway which belongs to lipid metabolism. According to the database of KEGG, four *NtADH* genes (*NtADH20*, *NtADH24*, *NtADH48* and *NtADH51*) are involved in the pathway of alpha-linolenic acid metabolism, and the contents of 2 metabolites in this pathway, namely 9-hydroxy-12-oxo-10(E), 15(Z)-octadecadienoic acid and 9-hydroxy-12-oxo-15(Z)-octadecenoic acid were significantly up-regulated during the curing process. Based on the qRT-PR analysis, these 4 ADH genes (*NtADH20*, *NtADH24*, *NtADH48* and *NtADH51*) exhibited significant different expression level at the initiation of the curing process (Fig. 12).

## Discussion

ADH gene family members are widely distributed in eukaryotes and prokaryotes [17, 25]. With the development of genome sequencing technology, a series of *ADH* genes or *ADH*-like genes have been identified in the genomes of Poaceae, Rosaceae, Brassicaceae, Fabaceae, and Pinaceae plants [26]. In this study, a total of 53 ADH genes identified in tobacco, including 5 long-chain genes, 1 short gene, and the remaining 47 belonging to the medium-chain ADH protein superfamily. This distribution is consistent with the typical abundance of *ADH* genes in most plant species [4]. It has been reported that polyploidization and gene region-specific duplication (tandem repeats and segmental repeats) are important mechanisms for the expansion of plant gene families [27]. Phylogenetic analysis showed that certain subfamilies only included *ADH* genes from tobacco, which indicated the special characteristics of these gene clusters specific to the tobacco. In addition, some *ADH* members of the tobacco clustered with *ADH* genes from other species, indicating a shared ancestry before diverging through species differentiation. Three *ADH* homologous gene clusters and three pairs of collinear gene pairs were identified, representing that duplication events were the main source of expansion of the tobacco ADH gene family, potentially due to the allotetraploid nature of tobacco.

Generally, the evolution of gene families largely depends on the organization of gene structure. In this study, significant variations were observed in the nucleotide sequence lengths, exon/intron structure, and protein motif composition among the 53 *NtADHs*. These differences highlight the intricate nature of the tobacco genome and the functional diversity within the ADH gene family. According to previous study, the typical number of introns of plant *ADH* genes is 9 [1]. In tobacco, only 9 *NtADHs* contained 9 introns (*NtADH35/36/43/44/11/12/14/21/22*). The reinsertion of introns at the specific location of their loss is considered extremely impossible. Therefore, the genes with more complete intron are considered to reflect the structure of ancestors more closely [1]. Moreover, a total of 20 conserved motifs were identified in tobacco ADH family, and the *NtADH* members displayed variations in the types and quantities of the motifs, irrespective of whether they belong to the same subfamily or distinct subfamilies. However, all members exhibited the presence of both ADH_N and ADH_zinc_N motifs, indicating the conservation and diversity of ADH gene family in tobacco. In addition, ADH, TADH, PDH, and CAD families all belong to MDR superfamily [28], and the members of these family all rely on zinc cofactors for their functionality. The possibly reason may be the valence stability of zinc ligands that maintains the catalytic structure required by MDR proteins, and reduces the need for conservative amino acids. This phenomenon could potentially explain why zinc-containing MDR proteins typically tend to contain less conserved catalytic domains [28].

It has been reported that *HvADH1* in barley is an *S* gene and plays a pivotal role in regulating pathogen invasion [20]. Consequently, it is plausible to assume that *NtADH* genes clustered with *HvADH1* in the same subgroups might possess similar functions. The expression patterns of 13 selected *NtADH* genes were analyzed in response to *Ras* infection. The results revealed that 10 genes (*NtADH6/7/8/11/12/14/21/29/38/40*) exhibited significant up-regulation during the early stage (0–48 h) of infection. This finding suggests that the initial phase of infection plays a crucial role in the interaction between tobacco seedling and *Ras.* Furthermore, it infers that these *NtADH* genes may function as *S* genes, recognizing specific effectors and triggering a rapid immune response in tobacco through the invasion of abundant hyphae. In-depth investigation of these highly up-regulated *NtADH* genes would contribute to a better understanding of the interaction between tobacco and pathogen*.*

The members of ADH gene family exhibited numerous *cis*-acting regulatory elements in their promoters that associated with light-responsiveness phytohormones, defense signaling transduction and various stresses. During the process of topping, leaf senescence, *Ras* infection and hypoxia or anaerobic stress, these promoters play a significant role in regulating the expression of *NtADH* genes, thereby exert control over the growth and development of tobacco plants. Notably, four *NtADH* genes (*NtADH8/10/14/50*) exhibited high expression levels in both the topping process and during leaves senescence (Fig. 8). Furthermore, among them, *NtADH8*, *NtADH10*, and *NtADH14* displayed significant responses to *Ras* infection (Fig. 10), implying their crucial roles in the growth and development of tobacco plants. In addition, it was reported that plant ADH gene family plays an important role in responding to hypoxia or anaerobic conditions [1]. By searching the database of KEGG, four *NtADH* genes (*NtADH20*/*24/48*/*51*) clustered in subfamily F (Fig. 6) were found to be associated with the pathway of alpha-linolenic acid metabolism, and the contents of metabolites in this pathway were significantly up-regulated during the curing process (Fig. 12). Interestingly, these four *NtADH* genes (*NtADH20*/*24/48*/*51*) showed significant down-regulated under topping stress (Fig. 8b). Alpha-linolenic acid acts as an integral component in the growth and cellular metabolism processes of plants. It assumes that the dual roles as both a structural constituent and a metabolic modulator, carrying out essential functions in the regulation and organization of plant systems. Under stress factors, α-linolenic acid exerts its regulatory effect by modulating plant hormone signaling and the expression of related genes to regulating plant growth and stress tolerance [29]. The results indicate that the *NtADH* genes play important roles in responding to various stresses, and further investigation of these genes would significantly contribute to a better understanding of the function of ADH gene family in tobacco.

## Conclusions

In this study, a total of 53 *ADH* genes were identified in the genome of tobacco, which were categorized into 6 subfamilies. These *NtADH* genes were randomly distributed on 24 tobacco chromosomes. Among these genes, 5 *NtADH* gene pairs were originated from tandem repeats, and 3 pairs were originated from segmental duplications. The promoters of *NtADH* genes contained *cis*-regulatory elements associated with cell development, plant hormones, environmental stress, and light responsiveness. The expression levels of the ADH gene family varied at different stages of plant growth and development, and differential response were also found under various stresses. Four *NtADH* genes (*NtADH 20/24/48/51*) play essential roles in the regulation of alpha-linolenic acid metabolism pathway during hypoxia and high-temperature curing process in tobacco leaves. Our results provided valuable information for further functional study of *NtADH* genes in tobacco.

## Methods

### Identification of ADH gene family members in tobacco

A local whole-genome protein sequence database of tobacco was constructed based on the Solanaceae Genomics Network (https://solgenomics.net/) [30, 31], The HMM models ADH_N (PF08240) and ADH_zinc_N (PF00107) extracted from the Pfam database (http://pfam.xfam.org) were used as queries for retrieving the candidate ADH protein sequences in tobacco. The tool of BLASTP (E ≤ 1e^−10^) was used for the identification of ADH family members. The candidate protein sequence which contained conserved ADH domains (PF08240 and PF00107) was confirmed as the final ADH protein sequence based on the CDD program of NCBI (https://www.ncbi.nlm.nih.gov/cdd/) [32]. These *ADH* genes of tobacco were renamed (*NtADHs*). The physicochemical properties of the tobacco ADH proteins were predicted and analyzed using the ExPASy software (https://www.expasy.org), and the transmembrane topology structures were predicted using the TMHMM 2.0 and ABTMpro, and a list of *ADH* genes was constructed, which contained the corresponding gene IDs, gene chromosome localization information, and protein information.

### Gene structure and conserved motif analysis

Cluster X software [33] was used to perform multiple sequence alignment for the NtADH protein sequences, and the maximum likelihood method (ML) of MEGA-11 [34] software was used to generate the phylogenetic tree of tobacco NtADH protein family with the bootstrap value of 1000. The GFF format file of tobacco gene structure was obtained from Solanaceae genome database (https://solgenomics.net/) [30, 31], and the *NtADH* gene structure was analyzed based on the software Gene Structure Display Server (GSDS) (http://gsds.cbi.pku.edu.cn/) [35]. For conserved motif analysis, the MEME tool was used with the following parameters: the number of motifs was set to 20, and the width range of motifs was established to be 6–50 amino acids. The *cis*-regulatory elements in the promoter region (2000 bp upstream of the starting codon) of the ADH were identified by the online program of PlantCARE (http://bioinformatics.psb.ugent.be/webtools/plantcare/html/) [36].

### Chromosome localization and gene duplication

MapInspect software (https://mapinspect.software.informer.com) was applied to map the chromosomal positions of the *ADH* genes in tobacco. Based on the annotation information and the full genome protein sequences of tobacco [30], the MCScanX [37] software was used to analyze the possible segmental duplication and tandem duplication events with default parameters, and the TBtools software [38] was used for visualization. The tobacco K326 genome database released by Edwards (2017, Nitab-v4.5) was used as reference genome (https://solgenomics.net/ftp/genomes/Nicotiana_tabacum/edwards_et_al_2017/).

### Expression analysis of *NtADH* genes under conditions of leaf senescence and topping stresses

To examine the expression patterns of *NtADH* genes during leaf senescence, tobacco leaves at five maturity stages, namely M1, M2, M3, M4, and M5, were collected based on their visible appearance. The yellowing rates of the five stages increased gradually with the increase of maturity. The FPKM value of the *NtADH* genes at these five senescence stages of tobacco leaves were extracted from our recent RNA-Seq data (PRJNA772550) [39]. In addition, the expression profiles of the *NtADHs* were investigated under conditions of topping [11] based on the dataset of GSE153483. A map was generated using the heatmap function of the R gplots package [40].

### Expression analysis of *NtADH* genes in response to *Ralstonia solanacearum* L.

The tobacco variety of Hongda was cultivated using the floating seedling method. The tobacco seedlings were conventionally managed until the 3–5 leaf stage. A total of 75 tobacco seedlings were chosen and inoculated with a highly virulent pathogenic strain of *Ras* that had been isolated and maintained by our laboratory [41]. Inoculation was performed by mechanically wounding the roots and irrigating with 30 mL of *Ras* liquid with a concentration of 10^8^ cfu/mL. These plants were then cultured in a high-temperature and high-humidity greenhouse (30℃, approximately 80% humidity, 12 h/d light). Samples were collected at 0 h, 12 h, 24 h, 48 h and 96 h post-inoculation, with each biological sample consisting of 5 plants and a total of 3 replicates. For sampling, the seedlings were uprooted, and their root were quickly washed with sterile water to eliminate any attached soil and pathogens. These samples were immediately stored at − 80 °C for RNA extraction. Total RNA was extracted using the Hipure Plant RNA Mini Kit (Magen Biotech, Shanghai) and cDNA synthesis was performed using the SMART Kit (Takara). The expression levels of the *NtADH* genes were evaluated by conducting real-time quantitative PCR (qRT-PCR) using SYBR Green qPCR Premix (Low ROX), and the relative expression levels were calculated using the 2^−∆∆t^ method [42]. Three technical replicates were performed for each sample. The actin gene of tobacco was used as the internal reference gene, and the primers of *NtADH* genes (Additional file 8: Table S8) were designed using primer3 software (https://bioinfo.ut.ee/primer3-0.4.0/).

### Analysis of the metabolomics related to *NtADH* genes during curing process

To analyze the related metabolites of *NtADH* genes, the upper leaves of the tobacco variety Cui Bi No.1 (CB-1) grown in Yanping District, Nanping City, Fujian Province of China were used for the assessment. According to the curing process, the samples were collected at five key temperature stages during the curing process, including pre-yellowing stage (40℃) and post-yellowing stage (41℃), pre-fixation stage (43℃), fixation stage (45℃), and small cylindrical stage (46℃), and denoted as T1, T2, T3, T4, and T5, respectively. Each sample consisted of six leaves, and a total of 15 samples were collected with three biological replicates. The software Analyst 1.6.3 was used to process the mass spectrometry data. The metabolites that met the threshold of |Log2FC|≥ 1, VIP ≥ 1, and *P* < 0.05 were selected as differential metabolites. The KEGG (Kyoto Encyclopedia of Genes and Genomes) database was used to annotate the metabolites [43], the KEGG metabolic pathways associated with the differentially metabolites obtained from different temperature samples were analyzed (http://www.kegg.jp/kegg/pathway.html). The expression of *NtADH* genes before curing were analyzed using qRT-PCR, and the primers were list in the Additional file 8: Table S8.

### Supplementary Information

**Additional file 1: Table S1.** Syntenic analysis of ADH genes among tobacco, Arabidopsis and tomato.

**Additional file 2: Table S2.** Sequences of 20 predicted motifs of NtADH proteins.

**Additional file 3: Table S3.** a The accession number of ADHs from other plants in our paper. b Paralogous genes and orthologous genes of NtADH with other species

**Additional file 4: Table S4.** Cis-elements present in the promoters of NtADH genes.

**Additional file 5: Table S5.** The FPKM values of 53 NtADH genes at five senescence stages of tobacco leaves.

**Additional file 6: Table S6.** Metabolites of four comparison pairs in tobacco during hypoxia and high-temperature curing process.  

**Additional file 7. **The KEGG Enrichment.

**Additional file 8: Table S8.** Primers for real-time PCR.  

## Data Availability

The datasets generated and/or analysed during the current study are available in the NCBI Sequence Read Archive repository, https://www.ncbi.nlm.nih.gov/sra/PRJNA772550 and the Gene Expression Omnibus (GSE153483), https://www.ncbi.nlm.nih.gov/geo/query/acc.cgi?acc=GSE153483.

## Abbreviations

Ras: *Ralstonia**solanacearum* L.
Cyto: Cytoplasmic
MW: Molecular weight
pI: Isoelectric points
ML: Maximum likelihood
*NtADH*: ADH genes of N*icotiana**tabacum*
FPKM: Fragments Per Kilobase of transcript sequence per Millions base pairs sequenced
GSDS: Gene Structure Display Server
qRT-PCR: Quantitative real-time PCR
