# Supplementary material for: Genome-wide identification and expression analysis of the ADH gene family under diverse stresses in tobacco (Nicotiana tabacum L.)
Source: BMC Genomics. 2024 Jan 2;25:13. doi: 10.1186/s12864-023-09813-4 (PMC10759372; doi:10.1186/s12864-023-09813-4)
Supplement: Supplementary file 2 — Additional file 2: Table S2. Sequences of 20 predicted motifs of NtADH proteins. [file 12864_2023_9813_MOESM2_ESM.docx]

**Table S2** **Sequences of 20 predicted motifs of NtADH proteins**

| **Motif** | **Width** | **Motif Sequence** |
| --- | --- | --- |
| 1 | 29 | YPMVPGHEVVGIVEEVGSNVTKFKVGDKV |
| 2 | 29 | GLGGVGHMAVQFAKAFGAHVTVISTSNSK |
| 3 | 29 | GGYSEYTVVDZHFVVKIPENLPLEEAALL |
| 4 | 41 | AIGGMKETQEMLDFCAKHNITPDIEVVPMDYVNTALERLAK |
| 5 | 50 | KZEAJERLGADSFLVSSDPDQMQAAAGSLDGIIDTVSADHPLLPLJSLLK |
| 6 | 21 | DVRVKVLYTGJCHSDLHFWKG |
| 7 | 21 | CFVGSCRECENCKSGLENYCP |
| 8 | 21 | CAGITVYSPLKYFGLDKPGLH |
| 9 | 29 | PENEHPVKAFGWAARDTSGVLSPFKFSRR |
| 10 | 29 | GQVIRCKAAVAWEPGKPLVJEEVZVAPPQ |
| 11 | 27 | EJELDKFITHEVPFEEINKAFDLMLKG |
| 12 | 21 | EKAKKLGADEVJBYKTEEKPA |
| 13 | 35 | GKLKTVVDSKHPLSKAQDAWAKLEDGHATGKIILE |
| 14 | 21 | TDGGVDYSFECTGNVDTMJSA |
| 15 | 41 | QKYDAVVHCAKGIPWSTFEPNLSDTGKVIDLTPGPSAMCTY |
| 16 | 50 | NPLSALRMLEDFVTLKPGDTIVQNGATSIVGQCVIQLARLRGIHSINIIR |
| 17 | 26 | TLKGTLFGGYKPKSDLPSLVEKYLNK |
| 18 | 29 | RINTBRGVMJHDGQSRFSINGKPIYHFVG |
| 19 | 21 | GWGKLVLVGVPEKPLELPVFP |
| 20 | 50 | LGYBCVGGNAATLVLKLLKQGGTMVTYGGMSKEPITVSTSSFIFKDVTLH |
