# Supplementary material for: Genome-wide identification and expression analysis of the ADH gene family under diverse stresses in tobacco (Nicotiana tabacum L.)
Source: BMC Genomics. 2024 Jan 2;25:13. doi: 10.1186/s12864-023-09813-4 (PMC10759372; doi:10.1186/s12864-023-09813-4)
Supplement: Supplementary file 3 — Additional file 3: Table S3. a The accession number of ADHs from other plants in our paper. b Paralogous genes and orthologous genes of NtADH with other species [file 12864_2023_9813_MOESM3_ESM.zip › Additional file 3/Table S3a.docx]

**Table S3a The accession number of ADHs from other plants in our paper**

| Species | Genes | Accession number |
| --- | --- | --- |
| C*ucumis melo* L. | CmADH1 | MELO3C023685P4 |
|  | CmADH2 | MELO3C014897P1 |
|  | CmADH3 | MELO3C026552P1 |
|  | CmADH4 | MELO3C027151P1 |
|  | CmADH5 | MELO3C005792P1 |
|  | CmADH6 | MELO3C026553P1 |
|  | CmADH7 | MELO3C002189P1 |
|  | CmADH8 | MELO3C003251P1 |
|  | CmADH9 | MELO3C011043P1 |
|  | CmADH10 | MELO3C026554P2 |
|  | CmADH11 | MELO3C023687P1 |
|  | CmADH12 | MELO3C019503P1 |
|  | CmFDH1 | MELO3C022399P1 |
| *Solanum lycopersicum* L. | Le-ADH2 | NP_001234099.1 |
|  | Le-ADH3 | NP_001275080.1 |
|  | LeFDH | NP_001238796.1 |
|  | LeADH1 | XP_004238532.1 |
|  | LeADH5 | AAB33480.2 |
|  | LeADH7A | XP_004238409.1 |
|  | Le-ADHs | XP_004243555.1 |
| *Mangifera indica* L. | Mi-ADH1 | ADB43613.1 |
|  | Mi-ADH2 | ADB43614.1 |
| *Arabidopsis thaliana* L. | At-ADH1 | NP_177837.1 |
|  | AtADH7 | NP_199040.1 |
|  | AtFDH | CAA57973.1 |
|  | AtFDH1 | AAB06322.1 |
|  | AtADH4 | NP_176652.2 |
|  | AtADHL | AAM63235.1 |
|  | At-ADH | AAM65725.1 |
| *Prunusarmeniaca* L. | Pa-ADH1 | ABZ79222.1 |
| *Hordeum vulgare* L. | Hv-ADH1 | CAA30600.1 |
