# Supplementary figures and images for: Genome-wide identification and expression analysis of the ADH gene family under diverse stresses in tobacco (Nicotiana tabacum L.)
Source: BMC Genomics. 2024 Jan 2;25:13. doi: 10.1186/s12864-023-09813-4 (PMC10759372; doi:10.1186/s12864-023-09813-4)

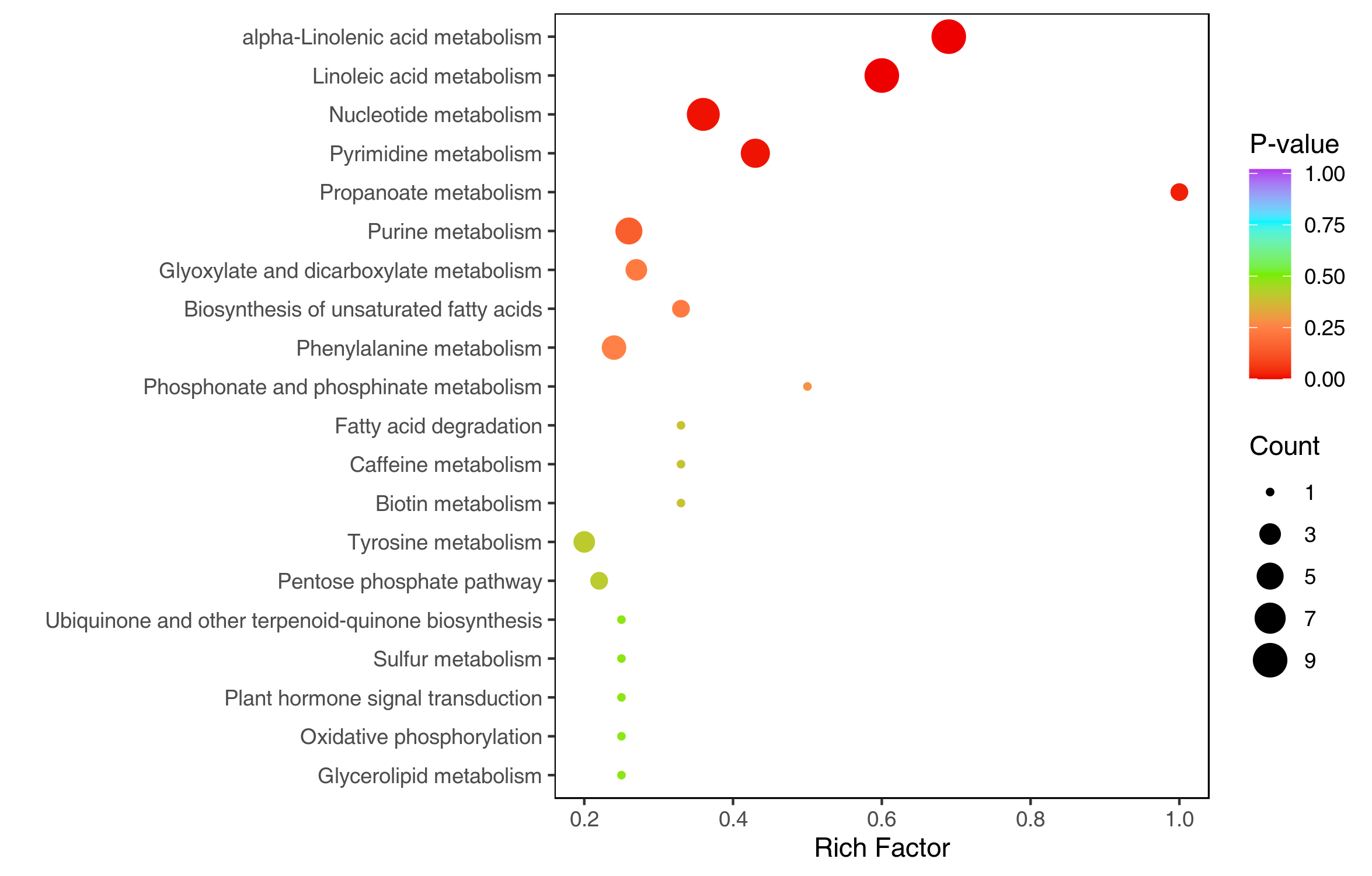

Supplement: Supplementary file 7 — Additional file 7. The KEGG Enrichment. [file 12864_2023_9813_MOESM7_ESM.zip › Additional file 7/Fig1 T1_vs_T2_KEGG_Enrichment.png]

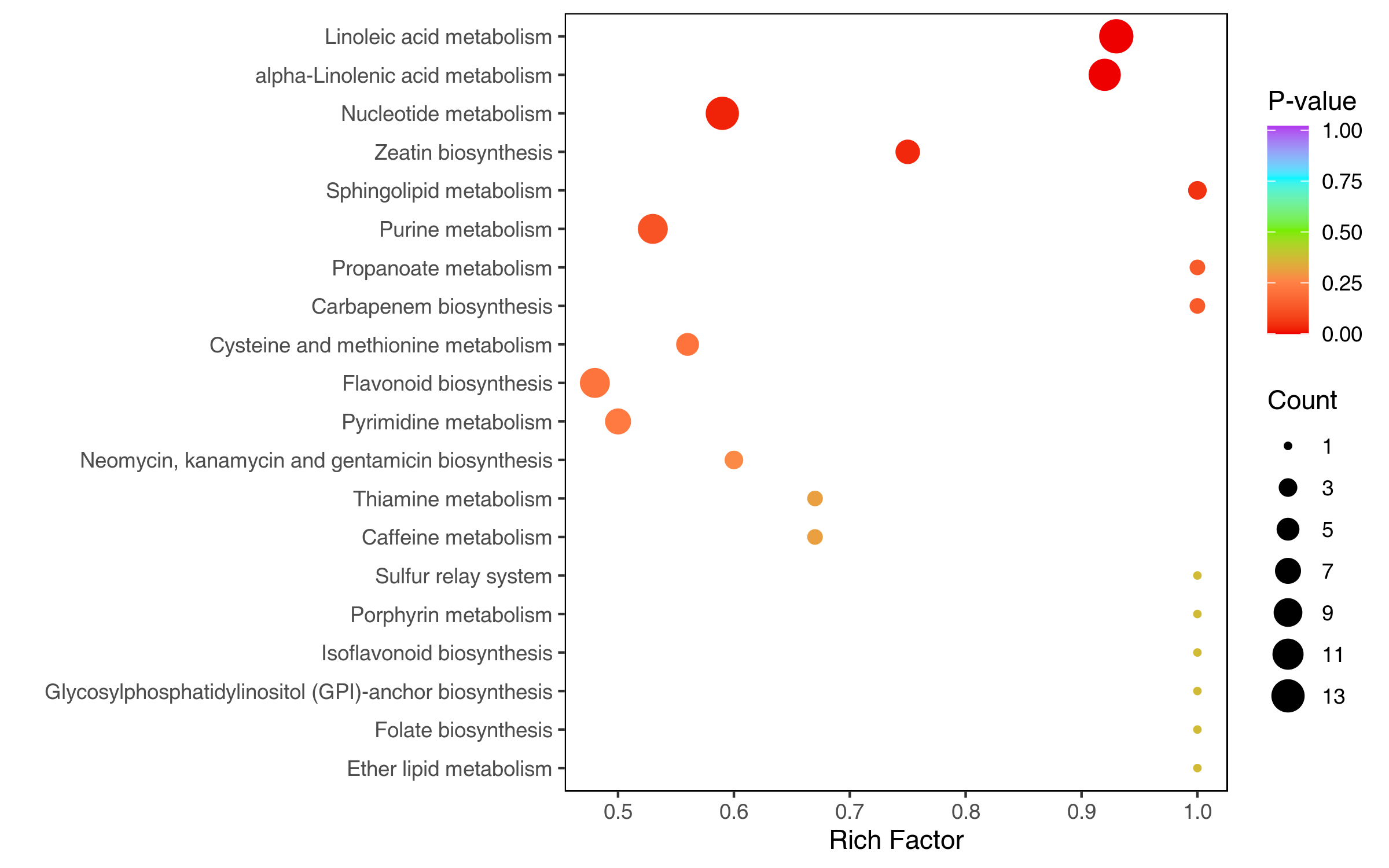

Supplement: Supplementary file 7 — Additional file 7. The KEGG Enrichment. [file 12864_2023_9813_MOESM7_ESM.zip › Additional file 7/Fig2 T1_vs_T3_KEGG_Enrichment.png]

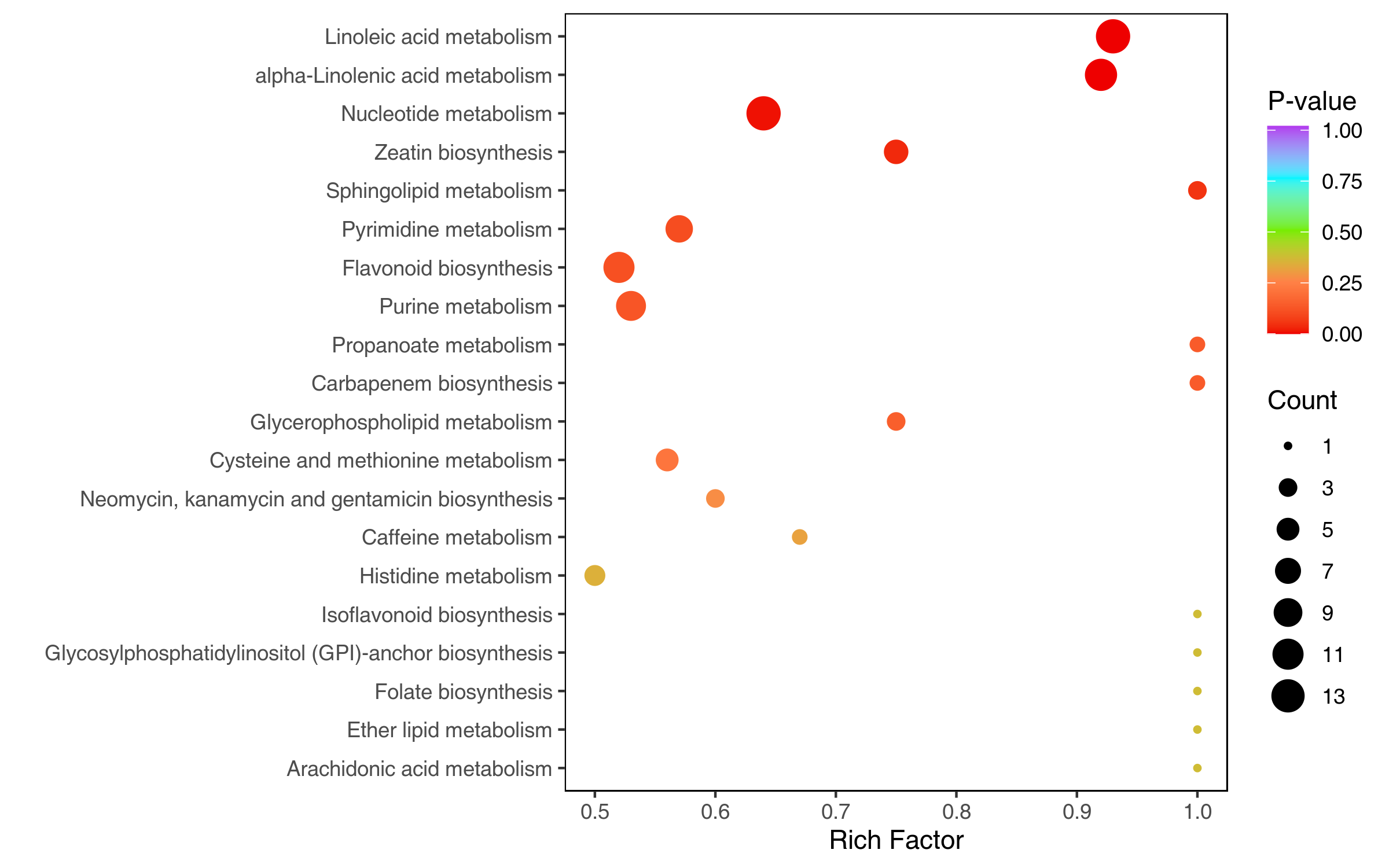

Supplement: Supplementary file 7 — Additional file 7. The KEGG Enrichment. [file 12864_2023_9813_MOESM7_ESM.zip › Additional file 7/Fig3 T1_vs_T4_KEGG_Enrichment.png]

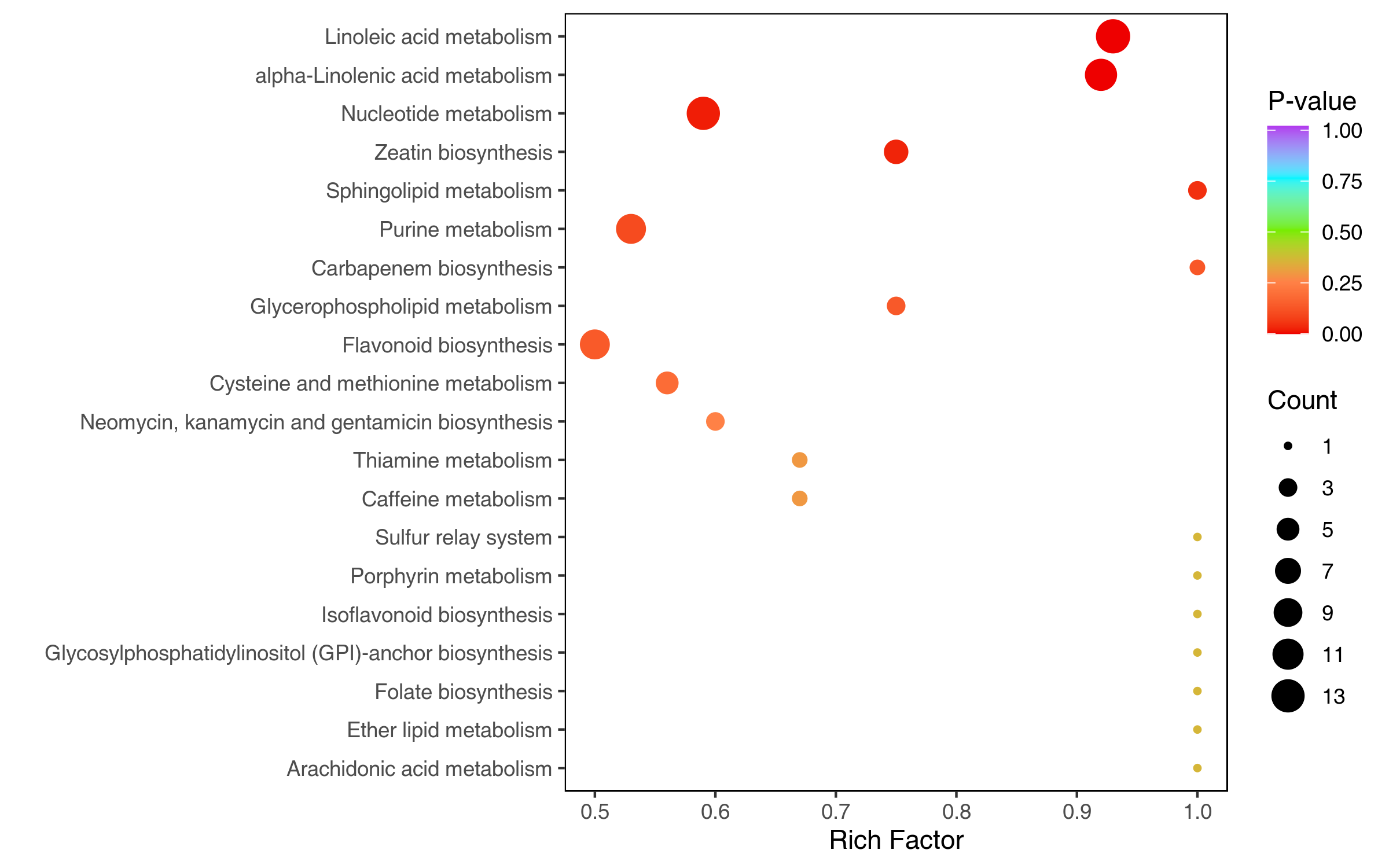

Supplement: Supplementary file 7 — Additional file 7. The KEGG Enrichment. [file 12864_2023_9813_MOESM7_ESM.zip › Additional file 7/Fig4 T1_vs_T5_KEGG_Enrichment.png]
